# Genomic Characterization of a Novel Tenericutes Bacterium from Deep-Sea Holothurian Intestine

**DOI:** 10.3390/microorganisms8121874

**Published:** 2020-11-27

**Authors:** Fang-Chao Zhu, Chun-Ang Lian, Li-Sheng He

**Affiliations:** 1 Institute of Deep-Sea Science and Engineering, Chinese Academy of Sciences, Sanya 572000, China; zhufc89@163.com (F.-C.Z.); lianca@idsse.ac.cn (C.-A.L.); 2 College of Earth and Planetary Sciences, University of Chinese Academy of Sciences, Beijing 101408, China

**Keywords:** deep-sea, holothurian, gut, symbiont, CRISPR, Tenericutes

## Abstract

Intestinal bacterial communities are highly relevant to the digestion, nutrition, growth, reproduction, and immunity of animals, but little is known about the composition and function of intestinal microbiota in deep-sea invertebrates. In this study, the intestinal microbiota of six holothurian *Molpadia musculus* were investigated, showing that their midguts were predominantly occupied by Izemoplasmatales bacteria. Using metagenomic sequencing, a draft genome of 1,822,181 bp was successfully recovered. After comparison with phylogenetically related bacteria, genes involved in saccharide usage and de novo nucleotide biosynthesis were reduced. However, a set of genes responsible for extracellular nucleoside utilization and 14 of 20 amino acid synthesis pathways were completely retained. Under oligotrophic condition, the gut-associated bacterium may make use of extracellular DNA for carbon and energy supplement, and may provide essential amino acids to the host. The clustered regularly interspaced short palindromic repeat (CRISPR) and restriction–modification (RM) systems presented in the genome may provide protection against invading viruses. A linear azol(in)e-containing peptide gene cluster for bacteriocin synthesize was also identified, which may inhibit the colonization and growth of harmful bacteria. Known virulence factors were not found by database searching. On the basis of its phylogenetic position and metabolic characteristics, we proposed that the bacterium represented a novel genus and a novel family within the Izemoplasmatales order and suggested it be named “*Candidatus* Bathyoplasma sp. NZ”. This was the first time describing host-associated Izemoplasmatales.

## 1. Introduction

Holothurians (Echinodermata: Holothuroidea) are marine invertebrates, presenting a wide vertical distribution from the intertidal zone to the deepest hadal trench. Usually, they dominate benthic megafaunal communities both in terms of quantity and biomass [1]. Epibenthic holothurians utilize organic matter in sediment, such as bacteria, microalgae, falling debris, and even some refractory components [2]. At the same time, they remix surface sediment and redistribute organic materials that arrive on the sea floor, due to their activities including food intake, burrow, and excretion [3]. Therefore, holothurians have important impacts on biotic nutrient recycling and energy flow to other benthic assemblages [4,5].

Deep-sea creatures mainly rely on the organic matter transported from the oceanic euphotic zone to acquire nutrition and energy. However, the majority of the organic matter is remineralized in the surface ocean, and globally, only around 10% of primary production is exported from the euphotic layer. Moreover, on average, 19% of exported organic matter can reach the ocean floor at 2000 m [6]. Organic debris is continuously decomposed and reused by various consumers during vertical landing and horizontal migration, and active organic matter such as proteinogenic amino acids, carbohydrates, and fatty acids are preferentially degraded, absorbed, and utilized [7]. This leads to refractory macromolecular materials (such as algaenan, cutin, suberin, and lignin that are derived from algae or higher plants) enriched in deep-sea sediments [3].

Studies have shown that gut microbes of holothurians may participate in the degradation of ingested organic materials. It has been reported that sediment microbiota through the digestive tract was selectively enriched by holothurians, along with an increase in genes related to carbohydrate and xenobiotics metabolisms in feces [8]. More direct evidence revealed a portion of cultured aerobic bacteria in the intestine of *Apostichopus japonicus* displaying various polysaccharide degradation activities [9,10]. In the intestine of holothurian *Molpadia musculus*, specific microbiota was constructed when organic matter was limited, and this specialized gut microbiota was thought to improve the host’s digestive efficiency [11].

In addition, the intestine and its associated microbiota have been proven to play a decisive role in strengthening the defense system and maintaining the proper function of intestinal epithelial cells for humans and other animals [12]. The intestinal bacteria of *Holothuria tubulosa* and *H. forskali* showed a range of antifungal and antimicrobial activities [13]. Moreover, probiotics isolated from holothurian intestines enhanced the host’s cellular and humoral immune response and improved intestinal microbiota homeostasis [14,15]. Intestinal microbiota may make a positive contribution to the holothurian’s survival and flourish in the hostile deep-sea environment. However, few research studies on intestinal microbiota of deep-sea holothurians have been reported. In our previous study, a highly reduced genome of *Spiroplasma* was obtained from the hindgut of a holothurian captured from the Mariana Trench. Despite its limited genome size, restriction–modification (RM) and clustered regularly interspaced short palindromic repeat (CRISPR) systems were obtained. As deposit feeders that directly filter sediment, the host holothurian might prevent virus invasion with the help of the *Spiroplasma* bacterium [16]. Even so, knowledge about the diversity, function, and forming mechanism of specialized intestinal microbiota in deep-sea holothurians is still limited.

In this study, we analyzed the gut microbial composition of bathyal *M. musculus*. This kind of holothurian is cosmopolitan in distribution, and moreover has a wide bathymetric range from shallow water to abyssal depth [17,18]. 16S amplicon sequencing revealed a significant increase in Tenericutes in their midguts. Then, by using metagenomic sequencing, we assembled a single draft genome, presenting a novel genus in the Izemoplasmatales order. Comparative genome analysis suggested it was a nucleoside degrader that worked as gut symbiont, providing essential amino acids to the host.

## 2. Materials and Methods

### 2.1. Ethics Approval

Specimens are provided by the NIWA Invertebrate Collection, National Institute of Water and Atmospheric Research (NIWA), Auckland, New Zealand, with the loan number 2017-003L.

### 2.2. Sample Dissection and DNA Extraction

Six sea cucumbers, *M. musculus*, were collected from the Bay of Plenty (two sites, namely BP1 and BP2) located off the northeastern coast of New Zealand’s North Island by trawling in April 2012 (Figure S1A). The detailed sampling information is listed in Table S1. Samples were immediately fixed with 99% ethanol onboard and stored in 80% ethanol for long-term preservation. Each specimen was dissected for its descending anterior intestine (foregut), ascending anterior intestine (midgut), and descending posterior intestine (hindgut) (Figure S1B) [19]. The intestinal contents were further separated and the tissues were rinsed in sterile water to remove as many residual contents as possible. Total DNA was extracted using a DNeasy PowerSoil Pro Kit (QIAGEN, Hilden, Germany). DNA concentration was determined with a NanoDrop 2000 (Thermo Fisher, Waltham, MA, USA).

### 2.3. 16S Amplicon Sequencing and Data Analysis

The V3-V4 regions of *16S rRNA* genes were amplified using barcode-tagged universal primers (341F: 5′-CCTAYGGGRBGCASCAG-3′; 802R: 5′-TACNVGGGTATCTAATCC-3′) and PrimeSTAR HS DNA polymerase (TAKARA, Dalian, China) [20]. The target gene fragments (approximately 460 bp) were separated by agarose gel electrophoresis and subsequently purified using a MinElute Gel Extraction Kit (QIAGEN, Hilden, Germany). The PCR products were sequenced using the Illumina Miseq PE250 platform (Personalbio Co., Shanghai, China).

Data analyses were performed using the plugins of QIIME2 v2018.6 [21]. Raw reads were demultiplexed according to the unique oligonucleotide barcodes ligated to 5′ end of forward primers. After removing adapter, barcode, and primer sequences using the cutadapt plugin, paired reads were joined with a minimum overlap length of 10 bp [22]. The merged sequences were filtered based on quality scores using the quality-filter plugin with default parameters [23]. Then, the sequences were denoised into amplicon sequence variants (ASVs) using the deblur plugin [24]. Taxonomic classification was performed by Vsearch global alignment, and the SILVA database version 132 was used as reference [25,26]. ASVs unassigned in kingdom level or classified as mitochondria, chloroplast, and eukaryota were filtered out. Relative abundance of specific taxa was statistically measured using the Mann–Whitney U test.

### 2.4. Metagenome Sequencing and Binning

Approximately 100 ng of total DNA extracted from the midgut of *M. musculus* sample BP1-2 were used for library preparation. High-throughput sequencing was performed with the Illumina Miseq PE250 platform. The quality of raw reads was checked by FastQC v0.10.1 (http://www.bioinformatics.babraham.ac.uk/projects/fastqc/). Raw reads were trimmed using Trimmomatic v0.36 with the following parameters: leading: 5, trailing: 5, slidingwindow: 4:15, and minlen: 50 [27]. Clean reads were further assembled into contigs using SPAdes-3.11 software, and the k-mer sizes were set as 21, 33, 55, 77, 99, and 127 [28]. Binning of draft genome was performed using MetaWRAP v1.2, in which contigs were processed in sequence as initial binning, bin refinement, and bin reassemble with the following parameters: three different algorithms MaxBin2, metaBAT2, and CONCOCT for metagenomic binning; contig length >2000 bp; completeness >50%; and contamination <10% [29]. Completeness and contamination of the metagenome-assembled genome (MAG) were determined by CheckM v1.1.1 with taxonomic-specific workflow [30].

### 2.5. Genome Annotation and Analysis

Five closely related species with available genomes were selected as references as follows: *Candidatus* (Ca.) Izemoplasma sp. HR1 (CP009415), Ca. Izemoplasma sp. HR2 (JRFF00000000), Ca. Izemoplasma acidinucleici (SDWO00000000), Ca. Izemoplasma sp. ZiA1 (NQYJ00000000), and *Haloplasma contractile* (AFNU00000000).

Genome annotation was processed in the same workflow. tRNA and rRNA genes were predicted by ARAGORN v1.2 and the RNAmmer 1.2 server, respectively [31,32]. Protein-coding sequences (CDSs) were identified by Prodigal v2.60 [33]. Subcellular localizations of translated proteins were predicted using PSORTb v3.0.2 [34]. Then, proteins were searched against the NCBI nr (download in 07/31/2019), COG, Pfam 32.0, and CAZy databases using Blastp v2.9.0+, with a maximum e-value cutoff of 1e-05 [35]. Kyoto Encyclopedia of Genes and Genomes (KEGG) annotation was performed by the KEGG Automatic Annotation Server (KAAS), with the bidirectional best-hit method and the representative genome set for prokaryotes [36]. CRISPRs and *cas* genes were predicted by the CRISPRCasFinder program [37]. Potential protospacers were predicted using the CRISPRTarget tool, by which a complement was counted if it had less than 4 mismatches and a protospacer-adjacent motif “NNNNGWWT” in the 3′ region [38,39]. AntiSMASH v5.0.0 was used for the prediction of secondary metabolite biosynthetic gene clusters [40]. Groups of orthologous proteins were clustered using OrthoFinder v2.3.8, in which the multiple sequence alignment (MSA) method was used for gene tree inference with the Muscle and IQ-Tree programs [41].

### 2.6. Phylogenetic Analysis Based on *16S rRNA* Genes

There were 143 sequences of the *16S rRNA* gene (>1200 bp) downloaded from the NCBI and SILVA databases for phylogenetic tree construction, including 107 Izemoplasmatales species. These sequences and the *16S rRNA* gene extracted from the MAG were aligned using MAFFT v7.427 and subsequently trimmed using trimAl v1.4 [42,43]. The TVMe+R6 model was selected by ModelFinder, and a maximum-likelihood phylogenetic tree was constructed using IQ-Tree v1.6.12 with 1000 ultrafast bootstraps [44].

### 2.7. Phylogenetic Analysis Based on Genome

In all, 49 reference genomes including 39 Tenericutes and 10 Firmicutes were downloaded from the NCBI database. Their phylogenetic marker genes were identified by AMPHORA2 [45]. Then, 21 marker genes shared by all genomes were selected for phylogenetic analysis as follows: ribosome-recycling factor; transcription termination/antitermination protein NusA; 50S ribosomal protein L1, L2, L3, L4, L5, L6, L11, L7/L12, L13, L14, L16, L27; 30S ribosomal protein S2, S3, S5, S9, S10, S19; and elongation factor Ts. Protein sequence alignments for individual genes were performed using Muscle 3.8.3 and trimmed with trimAl v1.4 [46]. The curated alignments were concatenated to generate an alignment length of 3503 amino acids. A phylogenomic tree was constructed using IQ-Tree with 1000 ultrafast bootstraps, and best-fit models for the 25 partitions were identified with ModelFinder. Taxonomic position of the MAG was also determined using the GTDB-Tk v1.0.1 tool [47].

### 2.8. Data Availability

Raw data of 16S amplicon sequencing and metagenome sequencing have been deposited at the NCBI Sequence Read Archive under the BioProject accession PRJNA628144. The *16S rRNA* gene sequence and draft genome of Ca. Bathyoplasma sp. NZ have been deposited at GenBank under the accession MT358871 and JABENI000000000, respectively.

## 3. Results

### 3.1. Composition of the Intestinal Microbiota

**Figure 1:**
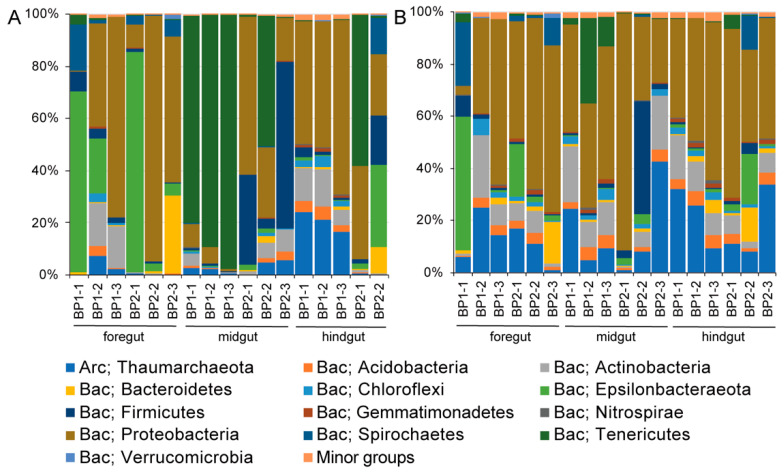
The bacterial composition in (**A**) the intestine and (**B**) its content of *Molpadia musculus*. The amplicon sequence variants (ASVs) are classified at the phylum level. BP1 and BP2 indicate two sampling sites in the Bay of Plenty. There are three individuals for each site. The hindgut of BP2-3 sample failed to amplify the *16S rRNA* gene fragments. A phylum with a percentage less than 1% is classified into minor groups. Bac, Bacteria; Arc, Archaea.

In total, 2,653,695 paired-end reads were generated for all intestine and content samples, with at least 50,026 reads for each one. After reads merging, quality filtering, and sequence denoising, 925,866 sequences were retained (Table S2). Furthermore, they resulted in 4019 ASVs that were classified into 35 phyla and 526 genera by taxonomic assignment. At the phylum level, Proteobacteria and Epsilonbacteraeota accounted for the vast majority of microorganisms in the foreguts and hindguts of *M. musculus*. However, there was a significant increase in the proportion of Tenericutes in the midgut microbiota (Mann–Whitney U test, *p* < 0.05) (Figure 1A). At the genus level, more than 95% of the Tenericutes were assigned as unclassified Izemoplasmatales (Figure S2). Correspondingly, a diverse group of Proteobacteria occupied throughout the intestinal contents (Figure 1B).

### 3.2. Assembly of Draft Genome

In metagenome sequencing, a total of 3.96 Gbp raw reads were generated for the midgut sample, and 2.72 Gbp were retained after quality control. Metagenomic assembly and binning resulted in a single high-quality population genome bin. Because the majority of reads originated from the host’s DNA, only about 55.8 Mbp (~2.05%) clean reads were strictly (no mismatches) mapped to the bin, resulting in a sequencing depth of 30×. The MAG contained 44 scaffolds without ambiguous bases, and had a total length of 1,822,181 bp. Similar with other Tenericutes, it was characterized with a low G+C content of 29.09%. Three rRNA (5S, 23S, and 16S rRNA), 30 tRNA, and 1584 protein-coding genes were identified in the genome, and the coding density was estimated to be 82.41%. By database searching, 1315, 1095, 1135, and 772 predicted proteins were successfully annotated in the NCBI nr, COG, Pfam, and KEGG databases. According to the presence or absence of 177 Tenericutes-specific marker genes, the genome was estimated with a completeness of 97.14% and a contamination of 1.90% (Table 1).

### 3.3. Taxonomic Classification

**Figure 2:**
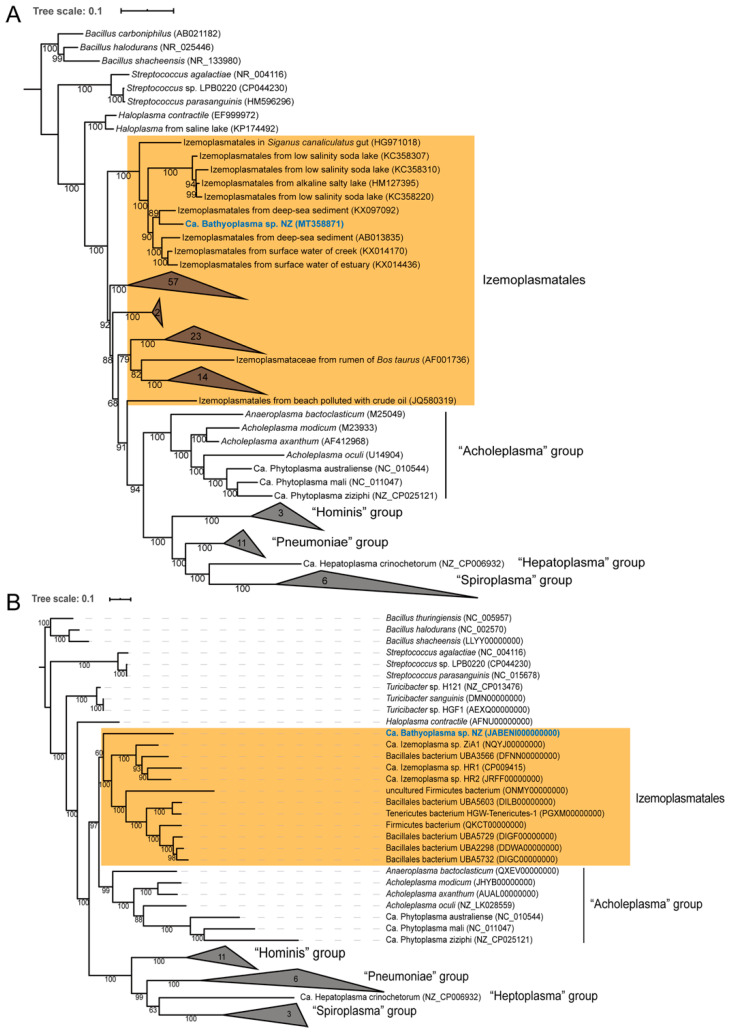
Maximum-likelihood phylogenetic tree based on (**A**) *16S rRNA* genes and (**B**) 21 concatenated conserved proteins. Three *Bacillus* (Firmicutes) bacteria are used as outgroups. The species in bold blue comes from this study. Bacteria belonging to the Izemoplasmatales order are masked by orange background. Bootstrap values are indicated on the branches, and accession numbers are labeled in parentheses. The species number in each collapsed node is also labeled. The tree scale bar represents the number of expected substitutions per site.

The full-length *16S rRNA* gene sequence was retrieved from the draft genome in order to determine its phylogenetic position in Tenericutes. By pairwise alignment, it shared 85.94%, 84.84%, 84.28%, and 87.27% identities with reported Ca. Izemoplasma sp. HR1, HR2, Ca. Izemoplasma acidinucleici, and *H. contractile*, respectively. In the 16S rRNA phylogenetic tree, the 16S rRNA sequence from this study was clustered with nine other uncultured bacteria from deep-sea sediment, salty lake, surface water, and white-spotted spinefoot *Siganus canaliculatus* gut, which were classified into the Izemoplasmatales order in the SILVA version 138 database. This clade was supported by a bootstrap value of 100%, and further separated with other groups of Izemoplasmatales (Figure 2A). The phylogenomic tree inferred from concatenated conserved proteins showed a similar topological structure. Between the Haloplasmas and Acholeplasmas group, the bacterium was placed as an independent sister branch of Ca. Izemoplasmas (Figure 2B).

Using the Genome Taxonomy Database (GTDB) for taxonomy analysis, the bacterium was identified as a member of the Bacilli class, with a relative evolutionary divergence (RED) value of 0.55. The RED value, reflecting lineage-specific rate of evolution, ranged from 0.378 to 0.578 at the class level [47]. It should be noted that Tenericutes were reclassified into the Bacilli class of Firmicutes in the GTDB. The sequence identity of the *16S rRNA* gene is also widely used for bacterial identification, and the proposed taxonomic threshold is 94.5% for genus, 86.5% for family, and 82.0% for order [48]. Due to its unique phylogenetic position, 16S rRNA sequence identity, and RED value, the bacterium was suggested to represent a novel genus and a novel family within the Izemoplasmatales order. The species name was temporarily designated as “Ca. Bathyoplasma sp. NZ” (Gr. adj. bathys, deep; -o-, connecting vowel; Gr. neut. n. plasma, something formed or molded. n. Bathyoplasma, a formed structure from deep ocean).

### 3.4. Genomic Comparison

Generally, the genome of Ca. Bathyoplasma sp. NZ (hereinafter referred to as BNZ) was comparable with Ca. Izemoplasmas in genome size and G + C content, but had fewer coding sequences. Like *H. contractile*, its coding density was sparser than Ca. Izemoplasmas. BNZ had the most mobile genetic elements (transposases, insertion sequences (ISs), and prophage), which probably hindered the completeness of genome assembly (Table 1). Among the six genomes, 2136 orthogroups were identified, including 504 ones shared by all species, while 341 genes (21.5%) were specific for BNZ (Figure S3).

**Figure 3:**
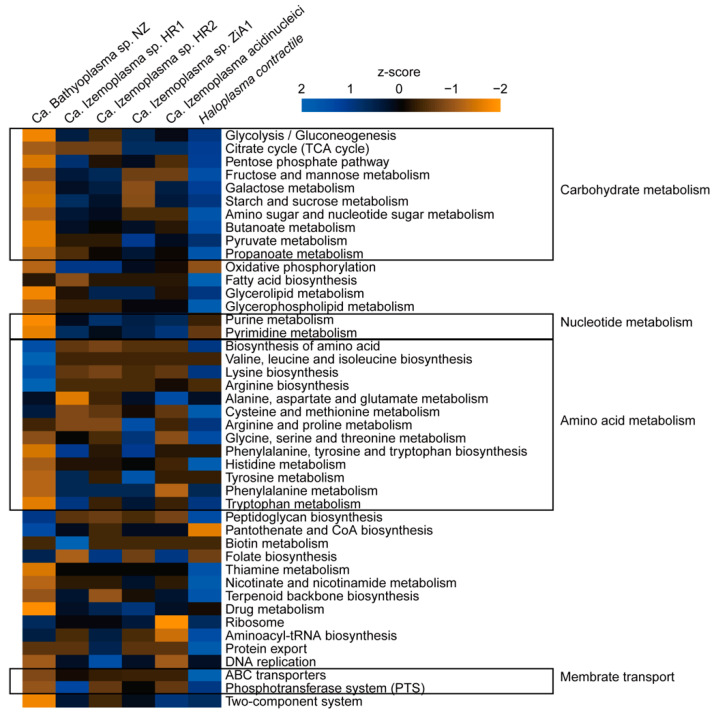
Comparison of gene numbers in different metabolic pathways. Gene numbers in each Kyoto Encyclopedia of Genes and Genomes (KEGG) pathway of BNZ and reference genomes are counted and normalized using z-score. Heatmap is generated using R.

**Figure 4:**
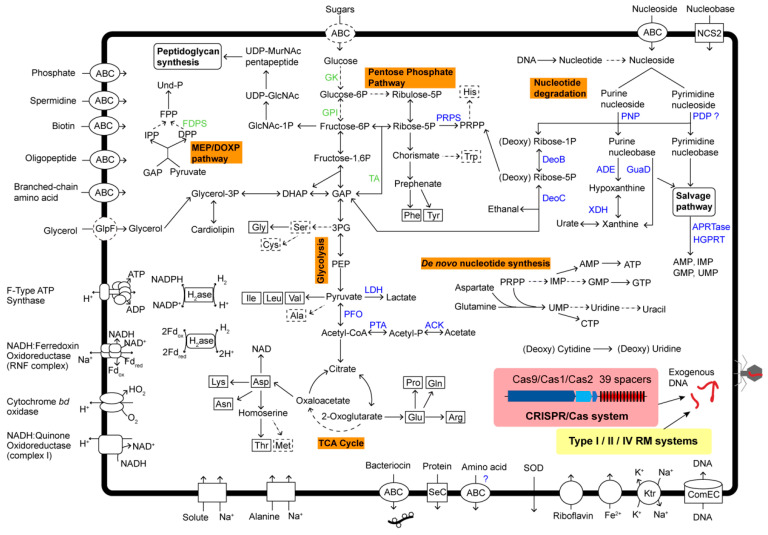
The predicted model of metabolism in Ca. Bathyoplasma sp. NZ. Absent elements of metabolic pathways and transporters are shown in dotted lines. Enzymes that were retained and lost in the genome of BNZ are presented in blue and green, respectively. Abbreviation: GAP, glyceraldehyde 3-phosphate; DHAP, dihydroxyacetone phosphate; 3PG, 3-phosphoglycerate; PEP, phosphoenolpyruvate; PRPP, phosphoribosyl pyrophosphate; IPP, isopentenyl diphosphate; FPP, farnesyl diphosphate; DPP, dimethylallyl diphosphate; Und-P, bactoprenol phosphate; GlcNAc, N-acetylglucosamine; MurNAc, N-acetylmuramic acid; GlpF, glycerol uptake facilitator protein; NCS2, nucleobase cation symporter-2; GK, glucokinase; GPI, glucose-6-phosphate isomerase; LDH, L-lactate dehydrogenase; PFO, pyruvate:ferredoxin (flavodoxin) oxidoreductase; PTA, phosphate acetyltransferase; ACK, acetate kinase; PNP, purine nucleoside phosphorylase; PDP, pyrimidine-nucleoside phosphorylase; ADE, adenine deaminase; GuaD, guanine deaminase; XDH, xanthine dehydrogenase/oxidase; DeoB, phosphopentomutase; DeoC, deoxyribose-phosphate aldolase; PRPS, ribose-phosphate pyrophosphokinase; CDD, cytidine deaminase; FDPS, farnesyl diphosphate synthase; APRTase, adenine phosphoribosyltransferase; HGPRT, hypoxanthine-guanine phosphoribosyltransferase.

According to the KEGG annotation results, genes involved in carbohydrate metabolism were notably reduced in BNZ, for example, starch and sucrose metabolism-related genes were entirely absent. ABC transporters were also reduced, especially for transmembrane transportation of simple sugars including glucose, fructose, galactose, maltose and trehalose, and phosphotransferase systems (PTSs) were completely missing. Conversely, amino acid biosynthesis pathways in BNZ were more complete than in the Ca. Izemoplasma species. The biosynthetic pathways for 14 kinds of amino acids were retained in BNZ, except alanine, serine, cysteine, methionine, histidine, and tryptophan (Figure 3 and Figure 4). Parts of genes associated with purine metabolism and pyrimidine metabolism were lost in BNZ. However, genes for basic cellular processes such as ribosome, aminoacyl-tRNA biosynthesis, and DNA replication were retained. Interestingly, the peptidoglycan biosynthesis associated-genes, which were usually absent in the wall-less Tenericutes, were discovered in BNZ (Figure 3).

### 3.5. Central Metabolic Pathways Inferred from BNZ

Metabolic pathways were reconstructed based on KEGG annotation. BNZ contained a relatively complete set of genes involved in the Embden–Meyerhof–Parnas glycolysis pathway and the nonoxidative branch of the pentose phosphate pathway, except for the lack of ADP-dependent glucokinase (GK), glucose-6-phosphate isomerase (GPI), and transaldolase (TA) (Figure 4). Pyruvate, the product of glycolysis, was reduced to lactate by lactate dehydrogenase (LDH) with concomitant oxidation of NADH. In another way, pyruvate could be further decarboxylated into acetyl-CoA by pyruvate:ferredoxin (flavodoxin) oxidoreductase (PFO). Then, through the phosphate acetyltransferase–acetate kinase (PTA-ACK) pathway, acetyl-CoA would be converted into acetate with acetyl phosphate as an intermediate product. BNZ did not possess a complete tricarboxylic acid (TCA) cycle, so lactate and acetate were probably the terminal products of heterolactic fermentation. A lipid carrier, bactoprenol phosphate (Und-P), essentially works in carrying peptidoglycan precursors through the cell membrane. Through the MEP/DOXP pathway, BNZ could produce its isoprenoid precursors, i.e., isopentenyl pyrophosphate (IPP) and dimethylallyl diphosphate (DPP), but lacked subsequent farnesyl diphosphate synthase (FDPS) for farnesyl diphosphate (FPP) synthesis.

Due to the absence of the arginine deiminase gene, BNZ could not generate ATP by arginine degradation. Similarly to Ca. Izemoplasma, BNZ contained a simplified electron transport chain containing NADH:quinone oxidoreductase (complex I), cytochrome *bd* oxidase, and NADH:ferredoxin oxidoreductase (RNF complex). It might produce ATP by substrate-level phosphorylation reactions or F-type ATP synthase.

More importantly, BNZ possessed a relatively complete nucleoside degradation pathway. A complex of general nucleoside ABC-type transport system (NupA/NupB/NupC/BmpA) and two copies of nucleobase cation symporter-2 (NCS2) family proteins were identified. Imported purine ribonucleoside (or deoxyribonucleoside) were broken down into purine nucleobase and D-ribose 1-phosphate (or 2-deoxy-D-ribose 1-phosphate) by purine-nucleoside phosphorylase (PNP). The 2-deoxy-D-ribose 1-phosphate produced D-glyceraldehyde 3-phosphate (GAP) for glycolysis, which was catalyzed by phosphopentomutase (DeoB) and deoxyribose-phosphate aldolase (DeoC) in turn. In the meantime, D-ribose 5-phosphate was converted into phosphoribosyl pyrophosphate (PRPP) by DeoB and ribose-phosphate pyrophosphokinase (PRPS). For the nucleobase components produced by PNP, they were further deaminized into hypoxanthine and xanthine, by adenine deaminase (ADE) and guanine deaminase (GuaD), respectively. All of these purine nucleobases were reused by the salvage pathway, which created adenosine monophosphate (AMP), inosine monophosphate (IMP), and guanosine monophosphate (GMP) with the catalysis of adenine phosphoribosyltransferase (APRTase) or hypoxanthine-guanine phosphoribosyltransferase (HGPRT). A pyrimidine-nucleoside phosphorylase (PDP) for the cleavage of pyrimidine nucleoside was also predicted in the genome, but it was truncated and only contained the PDP C-terminal domain. Meanwhile, BNZ lost the genes for de novo IMP and GMP production from PRPP, but reserved its capacity for cytidine triphosphate (CTP) biosynthesis.

### 3.6. Bacterial Defense System

A CRISPR/Cas system was identified in BNZ; it was composed of three *cas* genes (*cas9/cas2/cas1*) and a CRISPR locus with spacers of 30 bp and direct repeats of 36 bp (Figure 4). It belonged to the most streamlined type II-C1, based on the absence of additional *csn2* and *cas4* genes. Searching against phage, plasmid, and virus databases by using CRISPRTarget, no significant hits were returned for all 39 spacers. No complete CRISPR system was found in Ca. Izemoplasma or *Haloplasma* species. Another kind of bacterial defense system to fight against foreign DNA was also discovered in BNZ. Twenty proteins were predicted to be components of type I/II/IV RM systems (Table S3). Most of them were patchily distributed across the genome. Moreover, a DNA internalization-related competence protein ComEC/RecA involved in natural transformation of exogenous DNA was found. CRISPR and RM systems could target and degrade imported DNA, thus inhibiting natural transformation and virus invasion.

**Figure 5:**
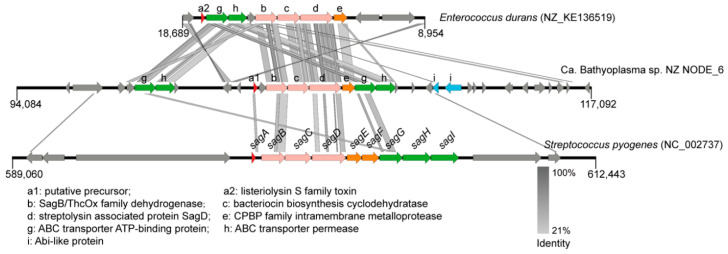
The linear azol(in)e-containing peptide synthesis gene cluster in Ca. Bathyoplasma sp. NZ. tBLASTx searches were performed against the listeriolysin S synthesis gene cluster in *Enterococcus durans* and the streptolysin O synthesis gene cluster in *Streptococcus pyogenes* with an e-value of 1e-5. Homologous regions are indicated with gray frames. The loci for the putative bacteriocin precursor, core enzymes, ABC transporter-related proteins, membrane-associated proteins, and other related proteins are shown in red, pink, green, orange, and blue, respectively. SagA~I, streptolysin associated protein A~I.

A gene cluster with a length of 23,010 nt for a novel linear azol(in)e-containing peptide biosynthesis was identified in BNZ (Figure 5). Three core biosynthetic genes encoded SagB/ThcOx family dehydrogenase, bacteriocin biosynthesis cyclodehydratase, and streptolysin associated protein SagD, respectively. The homologous gene cluster with highest similarity was found in *Enterococcus durans* ATCC 6056 (NZ_KE136519), which was responsible for listeriolysin S family toxin maturation and export.

## 4. Discussion

Tenericutes usually act as commensals or parasites of humans, animals, insects, and even plants [49]. Analysis of microbial community composition revealed that a kind of unclassified Izemoplasmatales bacterium flourished in the midgut cavity of *M. musculus*, but not in the content, which indicated a close relationship between this bacterium and the host. In order to localize the intestinal bacterium, formalin-fixed specimens were required for fluorescence in situ hybridization. In the present study, a representative draft genome was successfully recovered, which may help us uncover its potential role in the midgut of holothurian.

Extensive gene loss frequently occurs during the early stage of host adaptation, from a free-living to an intracellular lifestyle, as well as gene pseudogenization, duplication, recombination, and acquisition [50]. Compared with Ca. Izemoplasma, the gut-associated BNZ was featured with a more reduced genome. Specifically, the genes participating in carbohydrate usage and de novo nucleotide synthesis were diminished. Both Ca. Izemoplasma and *H. contractile* use a range of carbon sources, due to the presence of transporters responsible for sugar uptake [51,52]. However, these transporters were lost in BNZ, probably in order to couple with an ecological shift from seawater to intestinal habitats. Then, abundant IS elements in the BNZ genome implied a history of frequent horizontal gene transfer. As a result, transferred genes enlarged the bacterial metabolic capability in response to the changed environment [53,54]. The patchy distribution of orphan RM system components may also result from frequent horizontal gene transfer and genome rearrangement. A similar phenomenon was reported in an endosymbiont Ca. Endomicrobium trichonymphae, in which the RM system remnants were consistently associated with genome rearrangements [55]. Similarly to *H. contractile*, the peptidoglycan pathway was nearly complete in BNZ. However, in fact, a cell wall was not observed in *H. contractile* by electron microscopy [51]. Moreover, the capacity of bactoprenol synthesis in BNZ was deteriorated due to the absence of FDPS. This was a potential adaptive strategy of gut-associated Tenericutes in response to the transition from a free-living to a gut-associated lifestyle [56]. Up to now, all reported Ca. Izemoplasma and *Haloplasma* bacteria were derived from marine sediment. They lacked a host dependency and were considered to be free-living representatives [52,57,58,59]. This is the first time describing the dependent-living Izemoplasmatales in detail.

Much extracellular DNA is preserved in both the surface and subsurface of marine sediment layers [60], and it may act as a nutrient and energy repository in oligotrophic deep-sea environments providing essential elements such as carbon, nitrogen, and phosphorus [61]. Represented by Ca. Izemoplasma acidinucleici, members of the Izemoplasmatales order were identified to be active DNA degraders [58]. They encode multiple extracellular nucleases and extracellular nucleotidases for decomposition of DNA polymers outside the cell, and then use the liberated nucleosides as nutrient and energy source. Based on genomic analysis, BNZ also seemed to depend on extracellular DNA to grow. However, BNZ preferred to import and utilize extracellular nucleosides and nucleobases. Due to the absence of extracellular DNA degrading enzymes, nucleotide transporters, and intracellular nucleotidases, it could not directly make use of extracellular nucleotides and their polymers for nutrients. The available nucleosides and nucleobases used by BNZ may originate from extracellular DNA polymer digestion by the host or other bacteria in the gut. The phenomenon of using DNA as nutrient was discovered in *Escherichia coli* and *Bacillus subtilis*, and its significance was mainly embodied as a competitive survival strategy under oligotrophic conditions [62,63]. Moreover, the exogenous nucleobases could be important for the salvage pathway of BNZ, considering its incomplete de novo nucleotide synthesis pathways. In spite of its limited metabolic capacities, BNZ retained abundant genes involved in the biosynthesis of 14 amino acids. These synthesized amino acids may be transported to the host with the help of unknown transporters. In this way, the condition of nutritional imbalance or deficiency may be improved for holothurians. Furthermore, the genome of holothurian *Apostichopus japonicus* revealed that most amino acid pathways were missed in this kind of sea cucumber [64].

As a deposit-feeding invertebrate, deep-sea holothurians are challenged by numerous viruses and pathogens in the sediments [65]. CRISPR and RM systems may protect bacteria from invading genetic materials, such as bacteriophages and plasmids [16]. Moreover, the CRISPR system could also prevent natural transformation and, consequently, inhibited virulence acquisition [66]. It seems to be contradictory with the large amount of IS elements in BNZ. Actually, it has been reported that, on the one hand, exogenous DNA processed by restriction endonuclease is still able to recombine with a host’s chromosomal DNA with limited recombinant DNA fragment size [67]. On the other hand, the defense level of CRISPR and RM systems may be decayed, due to the relaxed pressure for maintaining these systems on the way to an intracellular lifestyle [68]. CRISPR systems were detected in 21 out of 52 mollicutes species, but they were relatively rare in main pathogens of humans, ruminants, and plants [69]. Prophage regions were detected in *Haloplasma* and three Ca. Izemoplasma species, reflecting their weaker resistance to virus, which may be caused by the lack of the CRISPR system. Linear azol(in)e-containing peptides are short, toxic peptides produced by specific bacterium that can inhibit the colonization and growth of other species. Although some linear azol(in)e-containing peptides acted as toxins, such as listeriolysin S and streptolysin O, the predicted precursor peptide in this study showed no similarity with validated toxins. Furthermore, by searching against the virulence factor database using a reported method, we confirmed no significant virulence factors encoded by the genome [70]. Moreover, bacteriocins secreted by gut microbiome could provide resistance against colonization by exogenous microorganisms, and maintain the community structure of gut microbiome in a health state [71].

## 5. Conclusions

The gut-associated Ca. Bathyoplasma sp. NZ was presented with characteristics of symbiotic bacteria, such as reduced genome and abundant IS elements. Genes encoded for simple sugar utilization and de novo nucleotide synthesis were reduced, while a mass of genes for nucleoside degradation and amino acid biosynthesis were presented in the genome. It may feed on extracellular nucleosides for energy and nutrition, and provide essential amino acids to the host. CRISPR and RM systems as well as secreted linear azol(in)e-containing peptides may protect the host from invading viruses and pathogens, thereby guaranteeing its survival in the harsh deep-sea environment.

## Figures and Tables

**Table 1:** General characteristics of genomes.

	Ca. Bathyoplasma sp. NZ ^a^	Ca. Izemoplasma sp. HR1	Ca. Izemoplasma sp. HR2	Ca. Izemoplasma sp. ZiA1	Ca. Izemoplasma acidinucleici	*Haloplasma contractile*
Habitat	intestine	methane seep sediment	methane seep sediment	tidal flat sediment	marine sediment	brine–sediment interface
Genome size (bp)	1,822,181	1,878,735	2,115,618	1,884,011	1,925,284	3,404,505
G+C content (%)	29.04	31.34	29.22	29.56	32.08	32.29
No. of scaffolds	44	1	78	34	191	34
No. of proteins	1584	1795	2222	1828	1927	3035
No. of rRNA	3	4	3	0	3	4
No. of tRNA	30	38	58	34	28	27
Coding density ^b^ (%)	82.41	91.60	90.14	92.20	91.90	82.16
Completeness ^b^ (%)	97.41	99.05	95.87	99.05	87.77	100
Contamination ^b^ (%)	1.90	2.86	6.67	5.71	5.88	2.86
No. of transposases	13	2	10	2	1	21
No. of IS elements	33	4	16	6	9	12
No. of prophage regions	0	0	2	1	1	1
Accession	JABENI000000000	CP009415	JRFF00000000	NQYJ00000000	SDWO00000000	AFNU00000000

^a^, the genome comes from this study; ^b^, the data are calculated by CheckM.

## Supplementary Materials

The following are available online at https://www.mdpi.com/2076-2607/8/12/1874/s1, Figure S1: Photograph of (A) the sampling sites and (B) the intestine of *M. musculus*. The samples were collected from the Bay of Plenty (BP) of New Zealand. Figure S2: Gut microbial composition in (A) the intestines and (B) its content of *M. musculus* displayed at the genus level. BP1 and BP2 indicate two sampling sites in the Bay of Plenty. There are three individuals for each site. The hindgut of the BP2-3 sample failed to amplify the *16S rRNA* gene fragments. The phyla in the classification are abbreviated by the first three letters. Genus with relative abundance <1% is categorized into minor groups. Figure S3: Venn diagram of orthologous groups among Ca. Bathyoplasma sp. NZ, *Haloplasma contractile*, and four Ca. Izemoplasma bacteria. The numbers in parenthesis indicate the number and percentage of proteins clustered in orthologous groups for each species. Table S1: Information of sampling sites. Table S2: Summary of 16S rRNA amplicon reads. Table S3: Predicted proteins associated with restriction–modification systems.
