# Supplementary material for: Genomic Characterization of a Novel Tenericutes Bacterium from Deep-Sea Holothurian Intestine
Source: Microorganisms. 2020 Nov 27;8(12):1874. doi: 10.3390/microorganisms8121874 (PMC7761423; doi:10.3390/microorganisms8121874)
Supplement: Supplementary file 1 [file microorganisms-08-01874-s001.zip › Suppl Tables.pdf]

Table S1. Information of sampling sites.

Site	Region	Voyage	Locality	Habitat	Depth (m)	Date
BP1	Bay of Plenty	TAN1206	36.9240°S 176.9797°E; 36.9192°S 176.9792°E	Slope	1502.0-1493.0	16/04/2012
BP2	Bay of Plenty	TAN1206	37.2392°S 177.6403°E; 37.2418°S 177.6308°E	Slope	1505.0-1494.0	19/04/2012

Table S2. Summary of 16S rRNA amplicon reads.

ID*	Intestinal tissue		Intestinal content	
	Reads-raw	Reads-denoised	Reads-raw	Reads-denoised
BP1-1F	72,995	37,107	80,074	35,947
BP1-1M	87,961	45,311	66,842	20,973
BP1-1H	85,777	28,833	84,522	26,166
BP1-2F	69,906	21,814	70,959	16,994
BP1-2M	113,127	60,649	201,605	70,785
BP1-2H	93,895	23,070	95,232	20,424
BP1-3F	59,137	21,629	62,034	19,753
BP1-3M	75,246	36,649	91,074	28,948
BP1-3H	77,524	19,959	62,273	17,053
BP2-1F	64,402	34,369	69,118	21,839
BP2-1M	57,561	25,727	71,982	28,935
BP2-1H	64,414	21,796	54,897	19,494
BP2-2F	70,834	29,284	67,010	22,555
BP2-2M	67,151	26,176	67,912	23,313
BP2-2H	64,664	33,767	63,992	27,009
BP2-3F	50,026	11,844	64,033	18,038
BP2-3M	52,958	12,494	51,125	5003
BP2-3H	N/A	N/A	101,433	12,159
Total	1,227,578	490,478	1,426,117	435,388

*: Samples were collected from two different sites in the Bay of Plenty (BP1 and BP2, n=3); F, forehut; M, midgut; H, hindgut.

Table S3. Predicted proteins associated with restriction-modification systems.

Seq-ID	Length	Pfam	Nr annotation	Identity	E-value
NODE_1 _4	352	PF04313.14, PF13588.6	endonuclease [Bacillus]	54.47%	1e-124
NODE_1 _99	410	PF09563.10	LlaJI family restriction endonuclease [*Clostridium botulinum*]	36.27%	2e-72
NODE_2 _49	410	PF10117.9	McrBC 5-methylcytosine restriction subunit McrC [*Haloplasma contractile*]	41.11%	2e-98
NODE_4_1	227	PF01420.19	restriction endonuclease subunit S [*Clostridium perfringens*]	42.33%	3e-33
NODE_4 _2	514	PF02384.16, PF12161.8	type I restriction-modification system subunit M [*Candidatus* Izimaplasma sp. ZiA1]	73.68%	0
NODE_4_3	942	PF18766.1, PF12008.8, PF04851.15, PF04313.14, PF00270.29	type I restriction enzyme R protein [*Candidatus* Izimaplasma sp. HR2]	53.81%	0
NODE_4_19	455	PF02976.15	restriction endonuclease [Faecalibacterium]	43.20%	7e-130
NODE_4_20	407	PF00145.17	DNA (cytosine-5-)-methyltransferase [Erysipelotrichaceae bacterium MTC7]	56.87%	2e-162
NODE_4_29	108	PF02384.16	restriction endonuclease subunit M [Clostridiales bacterium]	46.73%	4e-23
NODE_5_13	393	PF01420.19	restriction endonuclease subunit S [*Bacteroides* sp. AM44-19]	42.57%	1e-89
NODE_7_51	350	PF10117.9	5-methylcytosine-specific restriction enzyme subunit McrC [*Sporanaerobacter* sp. PP17-6a]	67.52%	2e-173
NODE_7_65	308	PF02384.16	class I SAM-dependent methyltransferase [*Turicibacter*]	29.69%	1e-45
NODE_7_75	323	PF00145.17	DNA (cytosine-5-)-methyltransferase [*Bacillus* sp. ABP14]	57.81%	9e-125
NODE_12_54	427	PF01420.19	restriction endonuclease subunit S [*Staphylococcus*]	46.10%	9e-128
NODE_12_55	499	PF02384.16, PF12161.8	SAM-dependent DNA methyltransferase [*Bacillus toyonensis*]	70.63%	0
NODE_16_21	627	PF02384.16, PF12161.8	SAM-dependent DNA methyltransferase [*Sulfurospirillum multivorans*]	44.27%	2e-163
NODE_16_22	420	PF01420.19	restriction endonuclease subunit S [*Arcobacter thereius*]	38.62%	7e-88
NODE_16_23	128	PF04313.14	type I restriction enzyme, R subunit, partial [*Halanaerobium* sp.]	38.84%	4e-14
NODE_19_30	231	PF01420.19	restriction endonuclease subunit S [*Flavobacterium* sp. GSP6]	51.64%	3e-66
NODE_25_14	169	PF01420.19	restriction endonuclease subunit S [*Paeniclostridium sordellii*]	59.17%	2e-57
