# Supplementary figures and images for: Genomic Characterization of a Novel Tenericutes Bacterium from Deep-Sea Holothurian Intestine
Source: Microorganisms. 2020 Nov 27;8(12):1874. doi: 10.3390/microorganisms8121874 (PMC7761423; doi:10.3390/microorganisms8121874)

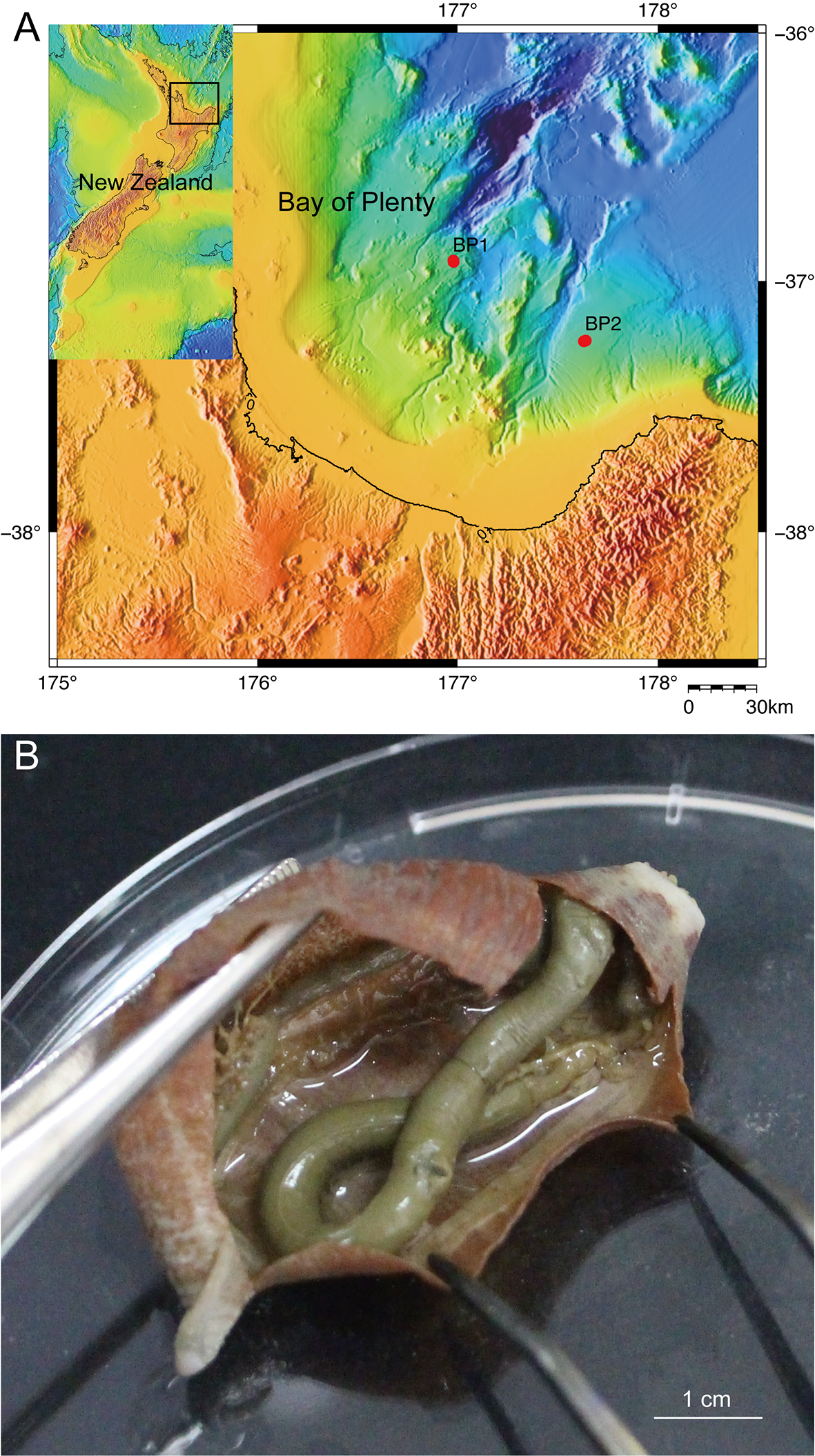

Supplement: Supplementary file 1 [file microorganisms-08-01874-s001.zip › Figure S1.tif]

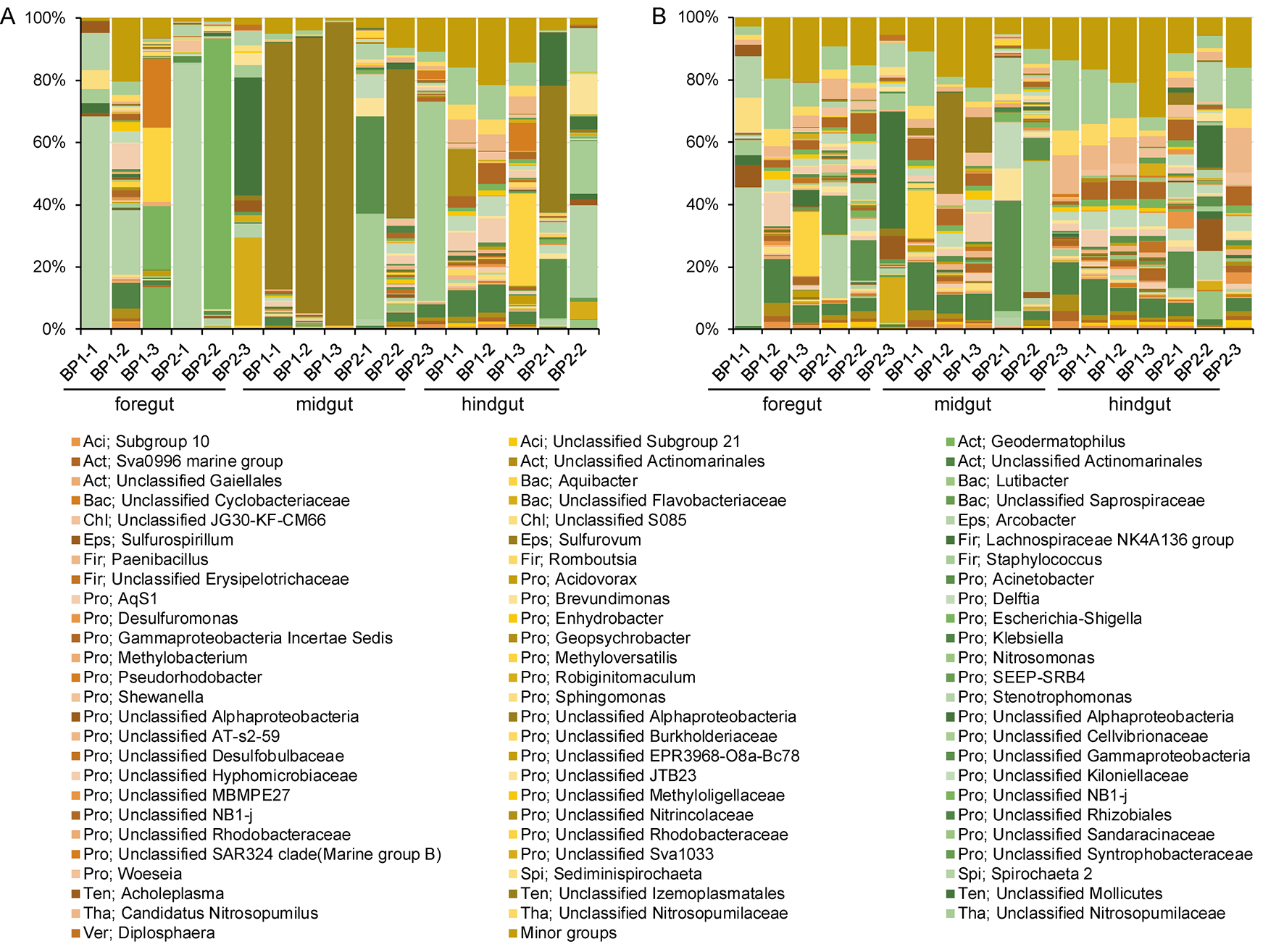

Supplement: Supplementary file 1 [file microorganisms-08-01874-s001.zip › Figure S2.tif]

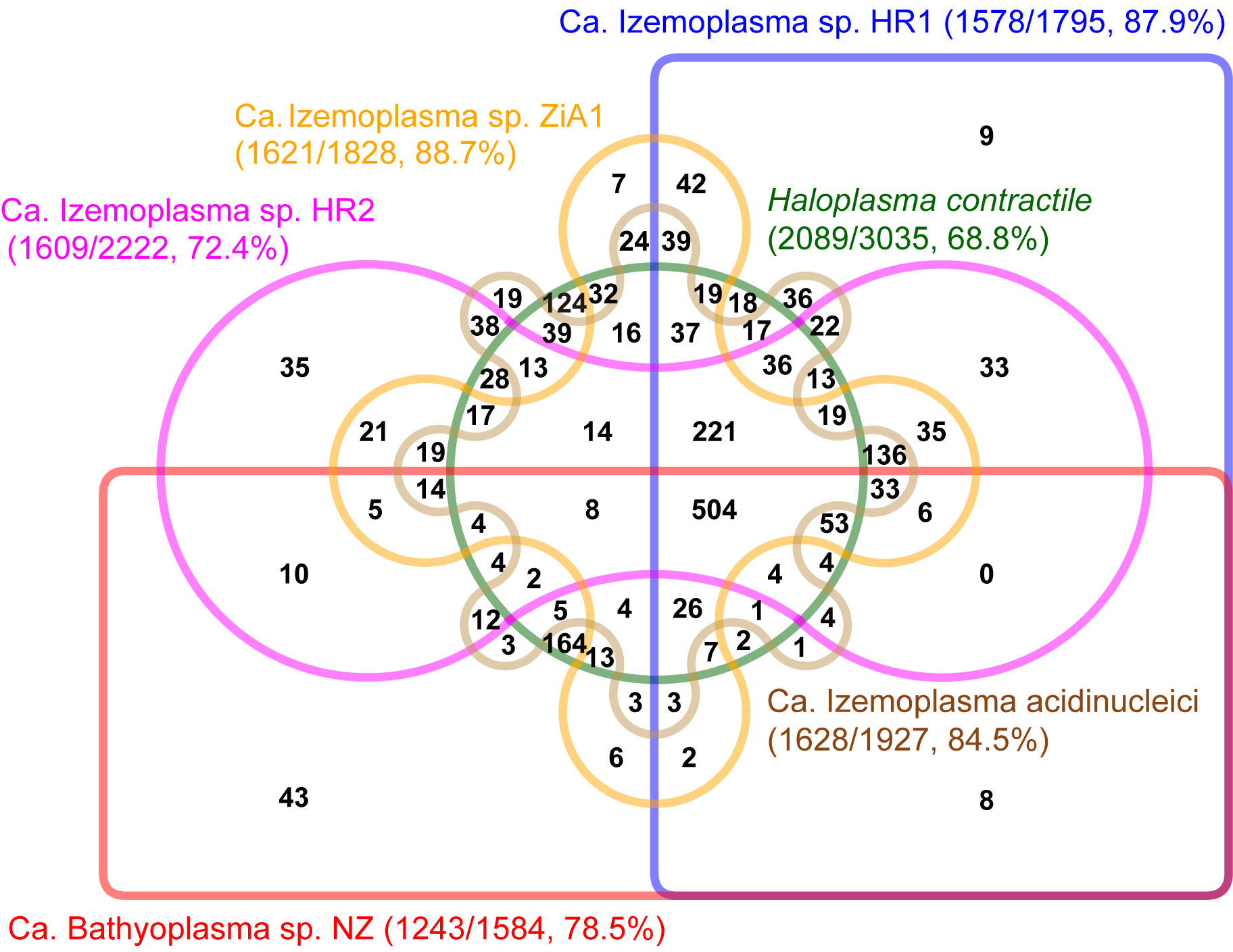

Supplement: Supplementary file 1 [file microorganisms-08-01874-s001.zip › Figure S3.tif]
